# Effectiveness of the MF59‐adjuvanted trivalent or quadrivalent seasonal influenza vaccine among adults 65 years of age or older, a systematic review and meta‐analysis

**DOI:** 10.1111/irv.12871

**Published:** 2021-06-03

**Authors:** Brenda L. Coleman, Ruth Sanderson, Mendel D. M. Haag, Ian McGovern

**Affiliations:** ^1^ Sinai Health Toronto ON Canada; ^2^ Dalla Lana School of Public Health Toronto ON Canada; ^3^ Western University London ON Canada; ^4^ Seqirus Netherlands B.V Amsterdam the Netherlands; ^5^ Seqirus Inc Cambridge MA USA

**Keywords:** MF59‐adjuvanted influenza vaccine, older adults, real‐world evidence, systematic literature review, vaccine effectiveness

## Abstract

**Background:**

Standard‐dose seasonal influenza vaccines often produce modest immunogenic responses in adults ≥65 years old. MF59 is intended to elicit a greater magnitude and increased breadth of immune response.

**Objective:**

To determine the effectiveness of seasonal MF59‐adjuvanted trivalent/quadrivalent influenza vaccine (aTIV/aQIV) relative to no vaccination or vaccination with standard or high‐dose egg‐based influenza vaccines among people ≥65 years old.

**Methods:**

Cochrane methodological standards and PRISMA‐P guidelines were followed. Real‐world evidence from non‐interventional studies published in peer‐reviewed journals and gray literature from 1997 through to July 15, 2020, including cluster‐randomized trials, were eligible. Two reviewers independently extracted data; risk of bias was assessed using the ROBINS‐I tool.

**Results:**

Twenty‐one studies conducted during the 2006/07–2019/20 influenza seasons were included in the qualitative review; 16 in the meta‐analyses. Meta‐analysis of test‐negative studies found that aTIV reduced medical encounters due to lab‐confirmed influenza with pooled estimates of 40.7% (95% CI: 21.9, 54.9; *I* ^2^ = 0%) for non‐emergency outpatient visits and 58.5% (40.7, 70.9; *I* ^2^ = 52.9%) for hospitalized patients. The pooled estimate of VE from case‐control studies was 51.3% (39.1, 61.1; *I* ^2^ = 0%) against influenza‐ or pneumonia‐related hospitalization. The pooled estimates for the relative VE of aTIV for the prevention of influenza‐related medical encounters were 13.9% (4.2, 23.5; *I* ^2^ = 95.9%) compared with TIV, 13.7% (3.1, 24.2; *I* ^2^ = 98.8%) compared with QIV, and 2.8% (−2.9, 8.5; *I* ^2^ = 94.5%) compared with HD‐TIV.

**Conclusions:**

Among adults ≥65 years, aTIV demonstrated significant absolute VE, improved relative VE compared to non‐adjuvanted standard‐dose TIV/QIV, and comparable relative VE to high‐dose TIV.

## 1 INTRODUCTION

It is estimated that the global disease burden from influenza includes up to a billion infections, 3–5 million cases of severe disease, and 290,000–650,000 deaths annually.^1^ Adults 65 years of age or older are particularly vulnerable to the complications resulting from influenza infections with higher rates of influenza‐associated complications and hospitalizations than younger people.^2^, ^3^, ^4^ Older adults make up the majority of influenza‐related deaths, with up to 90% of deaths occurring in this age group.^5^

Vaccination is the most effective method of preventing influenza infections and subsequent complications.^6^ The World Health Organization (WHO) recommends annual influenza vaccination for adults aged 65 or older.^7^ Vaccine efficacy has been estimated to be 60% in adults aged 18–65 years, but decreases in older age groups^8^, ^9^ and is influenced by virus, vaccine, and host‐related factors. A meta‐analysis of test‐negative design studies estimated vaccine effectiveness to be 51% for adults younger than 65 years of age compared to 37% for adults aged 65 years or older.^10^ In older adults, the natural biological aging process results in immunosenescence and a suboptimal immune response to vaccination in our most vulnerable population.^11^, ^12^ Rapid antigenic drift can occur resulting in an antigenic mismatch between the influenza strains included in a vaccine and the actual circulating strains.^13^ Strategies to improve the effectiveness of influenza vaccines among older adults have included the use of adjuvants or increased antigen content in the vaccine.

The squalene‐based adjuvant MF59® is an oil‐in‐water emulsion that has been shown to increase antigen uptake, macrophage recruitment, and lymph node migration and to broaden the spectrum of antibody recognition of hemagglutinin epitopes.^14^, ^15^ When added to seasonal influenza vaccines, MF59 elicits a greater magnitude and wider breadth of immune response to vaccination thereby improving protection compared to conventional inactivated influenza vaccines.^16^, ^17^ A MF59‐adjuvanted trivalent inactivated influenza vaccine (FLUAD™ [Seqirus]) was first approved for use in older adults in 1997 in Italy and was approved in 14 other European countries and 23 non‐European countries prior to its 2015 approval in the United States of America (USA). The quadrivalent formulation of FLUAD was first approved in Australia in 2019 and was approved in the USA and Europe in 2020.

In 2017, Domnich et al^18^ reported on the results of a systematic literature review and meta‐analysis of effectiveness of aTIV in people 60 years of age or older. The body of evidence on the vaccine effectiveness of aTIV in older adults has continued to evolve with additional studies being published since that review was conducted, particularly in recent seasons. This systematic review and meta‐analysis aim to identify and synthesize the available body of evidence to date.

The objective was to determine the effectiveness of seasonal MF59‐adjuvanted trivalent or quadrivalent seasonal influenza vaccine (aTIV/aQIV) relative to no vaccination or vaccination with standard or high‐dose egg‐based influenza vaccines among adults 65 years of age or older using real‐world evidence through a systematic review of the literature and meta‐analysis of comparable data.

## 2 METHODS

Cochrane methodological standards and preferred reporting items for systematic reviews and meta‐analysis protocols (PRISMA‐P) guidelines were employed. The protocol was registered with the international prospective register of systematic reviews, PROSPERO^19^ (CRD42020177747).

### 2.1 Eligibility criteria

Eligible study designs included prospective or retrospective non‐interventional cohort, case‐control, and test‐negative design studies. Cluster‐randomized controlled trials were eligible if the vaccine was assigned at the facility level leaving the vaccination of the individual at the discretion of the patient or attending healthcare provider. Reports published from the year of aTIV first's licensure (1997) through to the final search (July 15, 2020) were eligible for inclusion. Only original research was considered. Effect estimates based on meta‐analytic approaches were excluded. Information from peer‐reviewed journals as well as gray literature was included if it was available in English, French, Italian, or Spanish.

### 2.2 Information sources

An initial electronic search was conducted by a professional librarian in MEDLINE, Medline‐in‐Process, Medline EPUBS Ahead of Print, EMBASE Classic +EMBASE (OvidSP), Cochrane (Wiley), Web of Science (Clarivate Analytics), and Scopus (ScienceDirect) databases on April 9, 2020. The search was updated on July 15, 2020. Grey literature sources including European Network of Centres for Pharmacoepidemiology and Pharmacovigilance registry, Microsoft Academic, science.gov, Health Canada, Open Grey, Google Scholar, clinicaltrails.gov, European Union Clinical Trials register, and Cochrane central register of controlled trials were searched on April 13–15, 2020 and updated on July 15, 2020. Other sources of information included reports identified through hand searches, which included reviewing records suggested by the manufacturer and the reference lists of relevant articles.

### 2.3 Search

We used database subject terms and text words for FLUAD or influenza vaccines. The following search terms were included in the electronic databases: FLUAD terms (ie, fluad or MF59 or MF59 or aTIV or aQIV or chiromas or gripguard or Influpozzi Adiuvato or allV3 or allV4), influenza virus terms (ie, influenza vaccines, influenza, influenza*, flu, human, quadrivalent, influenza virus, influenza a virus, h1n1 subtype, h3n2 subtype, influenza b virus), vaccine terms (ie, vaccin* or immuni* or innoculat*), and types of vaccines (ie, adjuvant* or squalene* or emulsion*). Table S1 details the search strategy.

### 2.4 Study selection and data collection

Duplicate references were removed using EndNote^TM^ prior to review. Two reviewers independently assessed titles and abstracts to identify potential literature for full‐text review. The reviewers came to a consensus about eligibility when selections were discrepant. After downloading the available full‐text reports, the two reviewers conducted a review of the full text. Reference lists were search to identify other eligible reports.

The two reviewers independently extracted data using pre‐defined fields. Extractors came to agreement on the eligibility of reports and the data abstracted through consensus. Authors of abstracts were asked to provide posters, presentations, and publications of data presented at conferences/meetings. If more than one source was available, data from the more complete of the sources were extracted.

### 2.5 Data items

The exposure of interest was vaccination with MF59‐adjuvanted trivalent (aTIV) or quadrivalent seasonal influenza vaccine (aQIV) for the 1997/98 influenza season or later. The comparators of interest for absolute vaccine effectiveness included people who received either no influenza vaccine or a non‐influenza comparator vaccine that season. The comparators of interest for relative vaccine effectiveness included people who were vaccinated with egg‐based standard‐dose trivalent (TIV), standard‐dose quadrivalent (QIV), or high‐dose trivalent (HD‐TIV) influenza vaccine. The population included adults 65 years of age or older. There was no restriction on the setting and included people living in the community, residential facilities, or nursing homes.

The outcomes of interest were absolute and relative VE based on (1) laboratory‐confirmed influenza (any strain or by type/subtype/lineage) using molecular assays (eg, reverse transcription polymerase chain reaction (RT‐PCR), rapid molecular assays), viral culture or rapid cell culture, direct or indirect fluorescent antibody tests, or rapid influenza diagnostic tests; (2) non‐laboratory‐confirmed clinical influenza diagnosis based on diagnostic coding (ICD 9–11) or medical chart review for influenza, influenza in combination with other events such as pneumonia, or influenza antiviral prescription in combination with any of above; or (3) record of influenza‐related death. Influenza outcomes were specified by the clinical setting as applicable (ie, hospital, emergency department [ED], general practitioner/non‐ED outpatient [OP]). We sought outcome measures recorded ≥14 days after vaccination.

### 2.6 Risk of bias assessment

Risk of bias was assessed at the outcome level using the risk of bias in non‐randomized studies of interventions (ROBINS‐I) tool.^20^ Reviewers discussed discrepant results to reach consensus. The ROBINS‐I results were used to inform the overall assessment using the GRADE approach.^20^, ^21^ Funnel plots/Egger's test of bias was not used due to the paucity of similar studies^22^ (all meta‐analyses included <10 studies).

### 2.7 Synthesis of results

Random effects models were specified *a priori* for meta‐analyses in anticipation of heterogeneity in vaccine effectiveness due to viral, vaccine, and host factors. Heterogeneity was assessed using the *I* ^2^ statistic. Effect estimates were not pooled for <3 comparable studies nor for different study designs.^22^ Laboratory‐confirmed influenza and clinically diagnosed (not laboratory confirmed) influenza were not pooled due to differences in the sensitivity and specificity of these diagnostic criteria.

Separate meta‐analyses were conducted for absolute and relative VE. Odds ratio (OR) was pooled with relative risk (RR) and incidence rate ratio (IRR) when the rare disease assumption applied^23^ or if incidence‐density sampling was used in the study reporting the OR.

VE estimates based on medical encounters in different clinical settings (ie, hospital, ED, OP) were pooled for relative VE (only) if there were three or more studies that were otherwise comparable. VE estimates were pooled across seasons and countries to allow for the evaluation of general trends regardless of differences that may have impacted VE estimates. Only adjusted estimates were reported due to the potential for confounding and/or bias due to population differences.^24^ The primary adjustment method was used when specified by the authors; otherwise, data were pooled based on comparability of adjustment methods. Pooled estimates were reported regardless of high heterogeneity (ie, *I* ^2^ ≥ 75% and *p* < 0.05) but were investigated using subgroup analyses where possible. All pooled effect size estimates include 95% confidence intervals.

## 3 RESULTS

### 3.1 Study selection

The applied search criteria yielded a total of 6153 potentially relevant records. Duplicate records (*n* = 1779) were removed by the research librarian, and the titles and abstracts of the remaining 4367 records were screened for potential eligibility by two independent screeners. Seven additional records were identified through hand searches and included in the screening.

**FIGURE 1:**
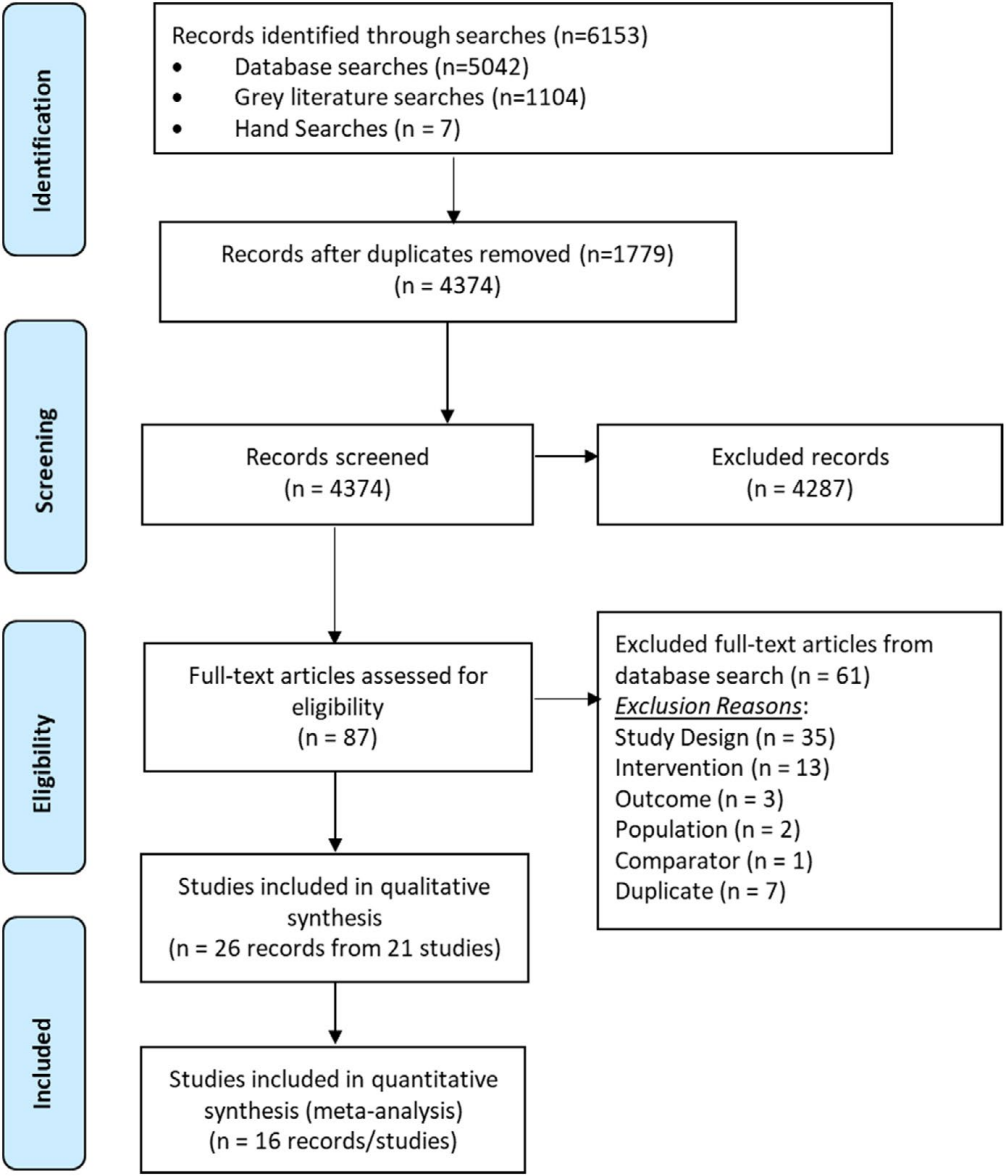
PRISMA flow diagram for literature review comparing the effectiveness of the MF59‐adjuvanted trivalent or quadrivalent seasonal influenza vaccine (FLUAD® or FLUAD® quadrivalent) for adults 65 years of age or older. PRISMA: Preferred reporting items for systematic review and meta‐analysis

The study identification, screening, and eligibility assessment process are summarized visually in the PRISMA diagram (Figure 1). Following the exclusion of 4287 records during title and abstract screening, 87 full‐text records were assessed for eligibility. Reports were excluded because they were review articles, systematic reviews, literature reviews, or meta‐analyses, the vaccine of interest (aTIV/aQIV) could not be separated from other vaccines or was not clearly delineated, they were a protocol or interim report, a duplicate record, an erratum to a record that already incorporated the changes, or the age criterion for participants was not strictly met. Twenty‐six records from 21 studies were deemed eligible for inclusion. Consensus was reached for all records selected by the reviewers.

### 3.2 Study characteristics

The 26 records included in the review covered 21 studies in total: seven test‐negative case‐control studies (with eight records),^25^, ^26^, ^27^, ^28^, ^29^, ^30^, ^31^, ^32^ five case‐control studies,^33^, ^34^, ^35^, ^36^, ^37^ eight cohort studies (with ten records),^38^, ^39^, ^40^, ^41^, ^42^, ^43^, ^44^, ^45^, ^46^, ^47^ and one cluster‐randomized trial (with three records).^48^, ^49^, ^50^ All studies were conducted in North America and Europe during the 2006/07–2019/20 influenza seasons. All test‐negative design studies reported absolute VE while two also reported relative VE against TIV/QIV. Case‐control studies evaluated the VE of aTIV compared to no vaccination (4/5 studies) and TIV (one study). Cohort studies evaluated the VE of aTIV compared to no vaccination (1/8 studies), TIV (5/8 studies), QIV (5/8 studies), and HD‐TIV (5/8 studies). A single cluster‐RCT evaluated relative VE of aTIV compared to TIV (Table S2). Absolute VE was not evaluated by any of the cohort studies or the cluster‐RCT. All studies are summarized below. Sixteen of the twenty‐one studies were included in at least one of the meta‐analyses.

### 3.3 Risk of bias

All studies except one were considered at moderate risk of bias using the ROBINS‐I tool (Table S3). One study was assessed as having a potentially serious risk of bias due to the selection of each control being conducted by the OP shortly after they identified each case.^35^

### 3.4 Effectiveness against laboratory‐confirmed influenza: test‐negative case‐control studies

Seven test‐negative case‐control studies conducted from the 2011/12 through to the 2019/20 season in the United Kingdom, Canada, and Italy used laboratory‐confirmed influenza for outcome ascertainment for people attending an OP^26^, ^28^, ^29^, ^30^, ^31^ (*n* = 5) or hospital with their illness^25^, ^27^, ^30^ (*n* = 3).

**FIGURE 2:**
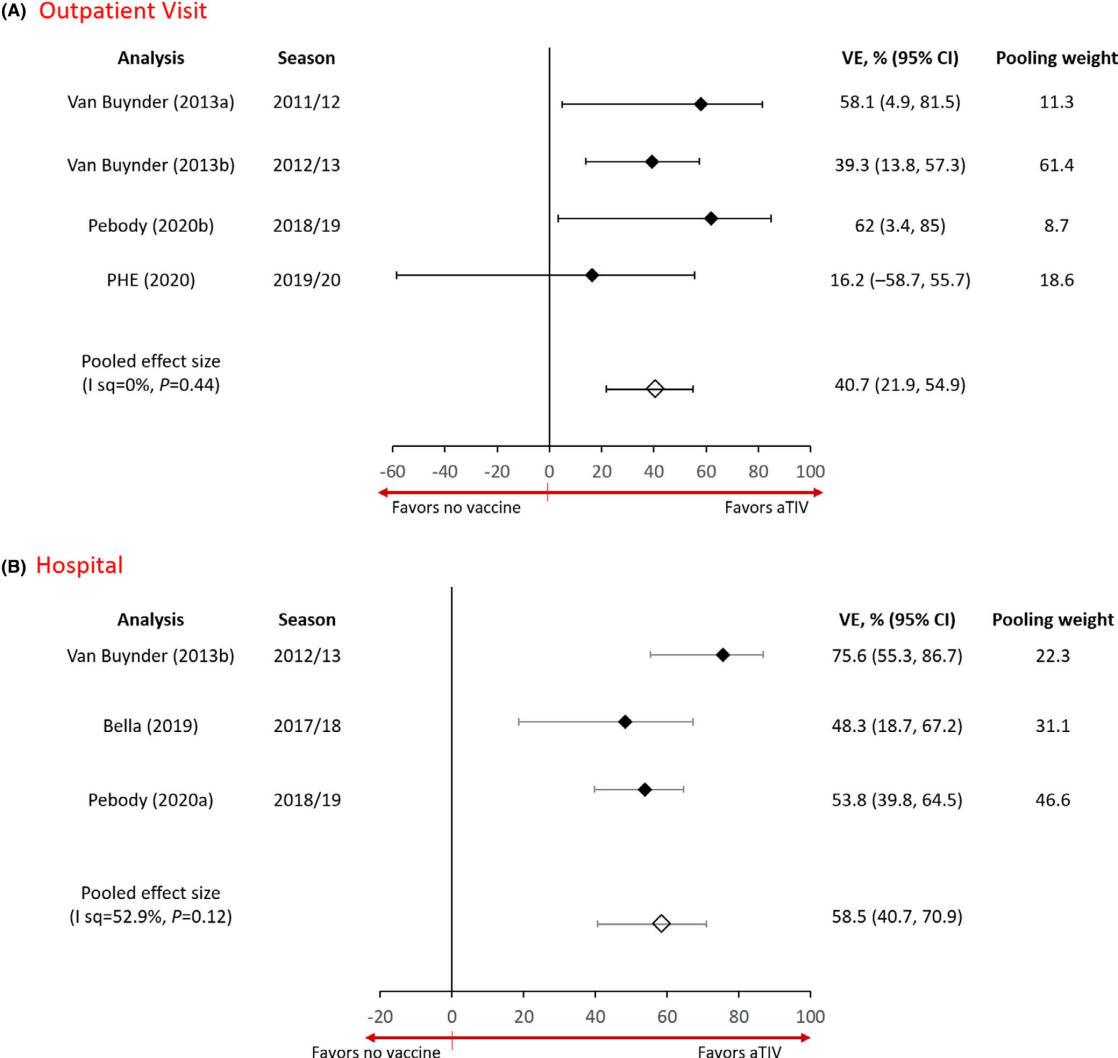
Forest plot of adjusted aTIV VE estimates for preventing (A) non‐emergency department outpatient visits or (B) laboratory‐confirmed influenza (any type/strain) in hospital patients. Adults 65 years or older, test‐negative design studies. VE: vaccine effectiveness; I sq, *I* ^2^. Pooling weight based on DerSimonian and Laird random‐effects meta‐analysis

Estimates from four studies that reported adjusted VE estimates for aTIV in preventing OP office visits due to lab‐confirmed influenza (any strain) ranged from 16.2% to 58.1%, with a pooled VE estimate of 40.7% (21.9, 54.9; (*I* ^2^ = 0%, *p*=0.44)^28^, ^29^, ^30^, ^31^ (Figure 2A). The fifth study reported VE against influenza A only (−1.1%).^26^ Estimates from the three studies that reported adjusted VE estimates for aTIV in hospitalized patients ranged from 48.3% to 75.6%, with a pooled estimate of 58.5% (40.7, 70.9; *I* ^2^ = 52.9%, *p* = 0.12)^25^, ^27^, ^30^ (Figure 2B). The results suggest that aTIV was effective in the prevention of laboratory‐confirmed influenza in both OP and hospital clinical settings.

### 3.5 Effectiveness against influenza illness, not laboratory‐confirmed: case‐control design

**FIGURE 3:**
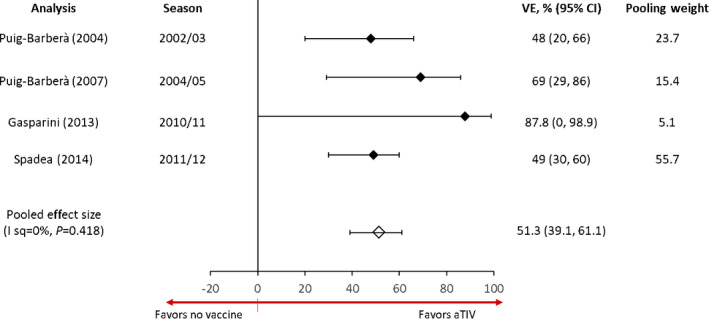
Forest plot of adjusted aTIV VE estimates for preventing influenza or pneumonia in hospitalized adults 65 years or older, case‐control design studies. I sq, *I* ^2^, VE: vaccine effectiveness. Pooling weight based on DerSimonian and Laird random‐effects meta‐analysis

Five case‐control studies conducted in Italy or Spain during the 2002/03 through 2016/17 influenza seasons were included. All of the studies evaluated cases diagnosed in a hospital setting with controls that were either hospital‐^33^, ^34^, ^36^ or community‐based.^35^, ^37^ One case‐control study^37^ was excluded from the meta‐analysis since the study pertained to a different outcome (pneumonia, stroke, or myocardial infarction) and comparator (TIV) than the other four studies. As shown in Figure 3, estimates from the four studies reporting VE estimates for aTIV in preventing hospitalization for influenza or pneumonia ranged from 48% to 87.8%, with a pooled estimate of 51.3% (39.1, 61.1; *I* ^2^ = 0, *p* = 0.42).^33^, ^34^, ^35^, ^36^

### 3.6 Effectiveness against influenza illness, not laboratory‐confirmed: cohort design

Eight studies used administrative data sources for medical care (clinic, ED, or hospital visits) to assess the effectiveness of aTIV relative to other influenza vaccines. Three of the studies were conducted in Italy using hospital catchment areas or OP rosters^38^, ^41^, ^42^ and five studies were conducted in the USA using Medicare^39^, ^40^ or other medical and pharmacy claims‐based information and medical records.^44^, ^45^, ^46^, ^47^, ^51^ These studies were conducted in the 2006/07 through 2018/19 influenza seasons. Six studies compared aTIV to TIV,^38^, ^39^, ^40^, ^41^, ^42^, ^44^, ^47^ five to QIV,^38^, ^39^, ^40^, ^44^, ^47^ and five to high‐dose TIV.^39^, ^40^, ^44^, ^47^, ^51^ Six of the eight studies reported on VE against medical encounters for influenza with or without pneumonia in various clinical settings including: OP; hospital or ED; or OP, hospital, or ED.

**FIGURE 4:**
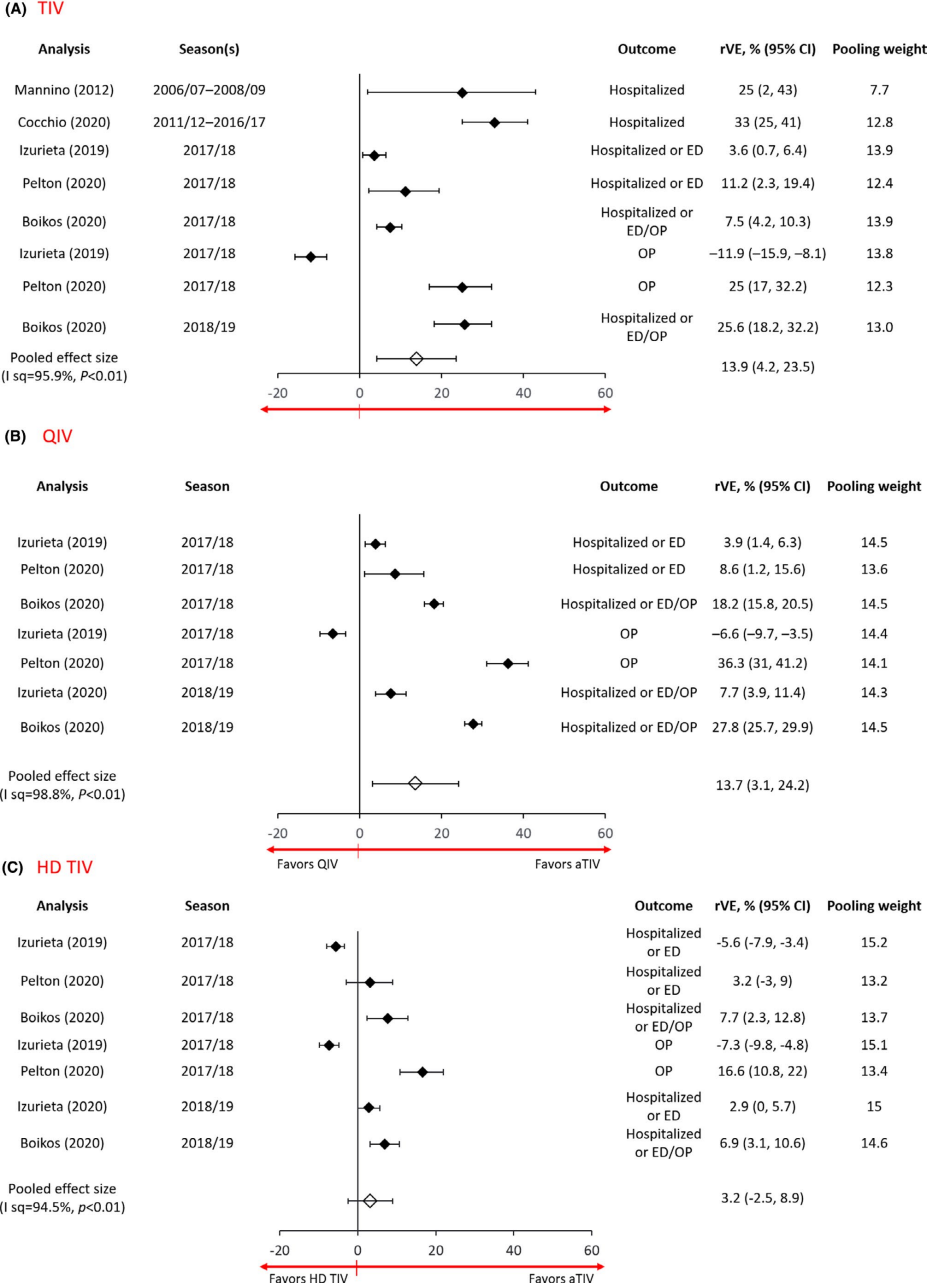
Forest plot of adjusted aTIV relative VE estimates compared with (A) standard‐dose TIV, (B) standard‐dose QIV, and (C) high‐dose TIV for preventing influenza with/without pneumonia. Adults 65 years or older, cohort study design studies (hospital, ED, or non‐ED outpatients). *Cocchio may have included some QIV in later seasons. aTIV, adjuvanted trivalent inactivated vaccine; ED, emergency department; GP, general practitioner; I sq, *I* ^2^; rVE, relative vaccine effectiveness; TIV, trivalent inactivated vaccine; VE, vaccine effectiveness. Pooling weight based on DerSimonian and Laird random‐effects meta‐analysis

The relative VE estimate for the prevention of influenza‐related medical encounters (hospitalization, ED visit, or OP visit) comparing aTIV to TIV ranged from −11.9% to 33%. As shown in Figure 4A, the pooled relative VE estimate showed a benefit of aTIV relative to TIV at 13.9% (4.2, 23.5) but with considerable heterogeneity (*I* ^2^ = 95.9%, *p* < 0.01).^39^, ^41^, ^42^, ^44^, ^47^ The relative VE of aTIV compared to QIV ranged from −6.6% to 36.3% with a pooled estimate of 13.7% (3.1, 24.2; *I* ^2^ = 98.8%, *p* < 0.01), indicating a benefit of aTIV over QIV^39^, ^40^, ^44^, ^47^ (Figure 4B). The relative VE comparing aTIV to HD‐TIV for reducing any medical encounters due to influenza and/or pneumonia ranged from −14.9% to 16.6% in five studies.^39^, ^40^, ^44^, ^47^, ^51^ The pooled estimate from four studies with similar outcomes was not different for aTIV compared with HD‐TIV at 3.2% (−2.5, 8.9), although there was considerable heterogeneity (*I* ^2^ = 94.5%, *p* < 0.01) between studies^39^, ^40^, ^44^, ^47^ (see Figure 4C). The Van Aalst^43^, ^51^ study was not included in the meta‐analysis since the outcome used (any respiratory condition ICD10: J) was broader than the outcome for others studies (influenza with or without pneumonia). In a sensitivity analysis, the relative VE of aTIV vs HD‐TIV remained non‐significant when the Van Aalst study was included in the meta‐analysis.

### 3.7 Cluster‐randomized trials

A cluster‐randomized trial of 823 nursing homes in the USA with almost 53,000 long‐term stay residents was conducted during the 2016/17 influenza season.^49^ Gravenstein et al estimated the relative VE of aTIV compared to TIV to be significantly higher for all‐cause hospitalization (6.0%; 1.0, 11.0) and hospitalization for influenza or pneumonia (21%; 4.0, 35.0) but not significantly different for hospitalization for all respiratory events (9%; −2.0, 19.0) or death (−5.0%; −11.0, 1.0). In a second analysis^48^ the adjusted relative VE of aTIV was 22% (1, 37) in preventing influenza‐related outbreaks at the facilities.

## 4 DISCUSSION

This systematic review includes information from 21 studies that compared the effectiveness of MF59 adjuvanted trivalent inactivated influenza vaccine (aTIV) to either no vaccination or standard or high‐dose egg‐based influenza vaccines for adults 65 years or older. All studies were conducted in North America or Europe during the 2006/07–2019/20 influenza seasons.

The results indicate that aTIV was effective against laboratory‐confirmed influenza, with pooled absolute VE estimates of 40.7% (21.9, 54.9) in reducing OP office visits and 58.5% (40.7, 70.9) in hospital patients using test‐negative case‐control designed studies. The individual VE estimates for aTIV against infection with any strain of influenza ranged from 16.2% in the 2019/20 season in the United Kingdom^31^ to 75.6% in the 2012/13 season in Canada.^30^ When estimates were pooled, the statistical measure of heterogeneity was considered low (for OP visits) to moderate (for hospitalizations).

In case‐control studies using influenza‐ or pneumonia‐related hospitalizations as the outcome, the pooled estimate of the VE of aTIV was 51.3% (39.1, 61.1). The individual estimates ranged from 48% to 88%^33^, ^34^, ^35^, ^36^ and there was low statistical heterogeneity in the studies that were conducted over four different influenza seasons in Italy and Spain.

In cohort design studies, the effectiveness of aTIV was significantly higher relative to standard‐dose TIV and QIV with pooled estimates of 13.9% (4.2, 23.5) and 13.7% (3.1, 24.2), respectively, for any influenza‐related medical encounter (OP, ED, or hospitalization). The pooled estimate of the relative VE of aTIV compared with HD‐TIV included the null and had a relatively narrow confidence interval around it (3.2%, −2.5, 8.9), indicating comparable ability in averting influenza‐related medical encounters. It is important, however, to note that the levels of heterogeneity were considerable in all pooled relative VE estimates.

Authors of three large cohort studies conducted in the USA in 2017/18 reported different results when comparing aTIV to other influenza vaccines in adults 65 years of age or older. The study conducted by Izurieta et al^39^ reported a relative advantage of TIV or QIV over aTIV, whereas the other two studies observed the opposite. This difference was only in the estimates from those seeking treatment from an OP visit (ie, not for hospital patients), a comparison not conducted in their 2018/19 study using the same methods.^40^ The source of the differences is unclear. Each study used similar outcomes and adjustment methods. However, they used different databases, each with their own specific characteristics and source populations. Izurieta et al studied non‐institutionalized Medicare beneficiaries of whom 40% had underlying heart disease, 30% had diabetes mellitus, and 45% were on home oxygen, for instance. In comparison, Pelton et al^44^ studied seniors using the IQVIA database of which about one‐third of the study population was insured by a third‐party payer. In their cohort, only 12% had heart disease, 20% had diabetes, and 4% were on home oxygen. Boikos et al used the Allscripts and Komodo databases, which, like Izurieta et al, included largely non‐institutionalized beneficiaries,^52^ but no other details about the baseline characteristics of the patients were available as the report was in poster format.

A general challenge and limitation of systematic reviews are the diversity in the methodological and clinical characteristics of the studies, which lead to myriad possible sources of variability in the effect size. The context of seasonal influenza adds additional complications due to virus‐related factors like antigenic drift of the circulating viruses and vaccine‐related factors like possible egg‐adaption, both of which can result in antigenic dissimilarity between circulating viruses and the vaccines, even by regions in the same country. Host‐related factors such as the effects of comorbidities, antigenic imprinting, and immunosenescence introduce additional sources of variability. Combined, these factors are likely contributors to variability in the individual point estimates for both absolute and relative VE. Pooling across multiple seasons with potentially distinct characteristics can introduce added variability, but allows for evaluation of the average effects over multiple seasons.

Despite careful considerations given in the current systematic review and meta‐analysis to achieve comparability of the pooled studies in terms of study design, comparators, and outcomes, high heterogeneity was still observed for the cohort studies. The possible sources of the heterogeneity were evaluated with the use of subgroup analyses by season and clinical setting. Subgroup analyses by clinical setting did not resolve the heterogeneity observed in analyses of the relative VE of aTIV compared with TIV or HD‐TIV, although it was reduced for QIV. Similarly, season‐specific subgroup analyses did not result in meaningful reductions in the heterogeneity, though this was only possible for the 2017–18 season. Further investigations of the sources of heterogeneity (eg, separating the analyses by both season and clinical setting) were not feasible due to the small number of studies in each subgroup. The analysis periods used by the different studies also have the potential to introduce additional variability. The studies by Izurieta et al,^39^ Cocchio et al,^42^ and Mannino et al^41^ restricted their analyses to peak periods of influenza activity, for example. In the absence of other potential sources of bias, it is expected that restricting to a period of higher influenza activity would increase the specificity of the outcome definition resulting in a more accurate, but potentially less precise, estimate due to reduction in sample size.^53^

It is likely that the differences in the statistical heterogeneity in meta‐analyses of absolute compared to relative VE analyses were at least partially caused by the available precision of the effect estimates. Meta‐analyses of absolute VE were only feasible for the test‐negative design and case‐control studies while meta‐analyses of relative VE were only feasible for the cohort studies. The test‐negative design and case‐control studies identified in this review generally produced less precise estimates, resulting in wider confidence intervals. Meanwhile, many of the cohort studies had very large patient populations and produced narrow confidence intervals. The more precise measurement of relative VE may highlight differences in the cohort studies in terms of study methods as well as virus, vaccine, and host‐related factors and their associated impact on vaccine effectiveness. Thus, the underlying variability in the effect size observed in the context of seasonal influenza coupled with precise estimates reported for the large cohort studies may explain the high heterogeneity observed in some of the meta‐analyses. However, high precision does not necessarily equate with high accuracy and therefore the observed heterogeneity could be the result of residual confounding, true variability in the underlying effects, or a combination of the two.

The use of random‐effects models for the meta‐analyses was specified *a priori* in anticipation of the different sources of heterogeneity described above. As future studies are published, additional subgroup analyses may become feasible and may allow for the use of additional methods like meta‐regression to better evaluate sources of heterogeneity. Future meta‐analyses could estimate the width of the underlying distribution with the use methods like Bayesian meta‐analysis which can generate a prediction interval that aims to estimate the width of the distribution.

## 5 CONCLUSIONS

The MF59‐adjuvanted trivalent influenza vaccine was effective in preventing influenza in adults 65 years of age or older. Compared to standard‐dose egg‐based QIV and TIV, aTIV was significantly more effective in preventing influenza‐related medical encounters (illnesses or hospitalizations). The effectiveness of aTIV was comparable to high‐dose TIV in preventing influenza‐related medical encounters. High heterogeneity was observed for all relative VE analyses with seasonal variation, distinct populations, and diverse outcomes potentially playing a role in the variability of the effect sizes coupled with precise effect estimates. As such, further research is needed to confirm the findings for relative VE.

## CONFLICT OF INTEREST

BLC and RS were employed by Sinai Health which received funding from Seqirus for this review. MDMH and IM are employed by Seqirus.

## AUTHOR CONTRIBUTIONS

**Brenda L Coleman:** Data curation (equal); Formal analysis (equal); Investigation (equal); Methodology (equal); Project administration (equal); Supervision (equal); Writing – original draft (equal); Writing – review and editing (equal). **Ruth Sanderson:** Validation (equal); Writing – original draft (equal); Writing – review and editing (equal). **Mendel Haag:** Conceptualization (equal); Funding acquisition (equal); Resources (equal); Writing – review and editing (equal). **Ian McGovern:** Conceptualization (equal); Funding acquisition (equal); Methodology (equal); Writing – review and editing (equal).

### PEER REVIEW

The peer review history for this article is available at https://publons.com/publon/10.1111/irv.12871.

## Supporting information

Table S1‐S3

## Data Availability

The data that support the findings of this study are available from the corresponding author upon reasonable request.

## References

[1] World Health Organization . Global influenza strategy 2019–2030. 2019.

[2] Rothberg MB . Complications of viral influenza. Am J Med. 2008;121(4):258‐264. 18374680 10.1016/j.amjmed.2007.10.040 PMC7172971

[3] Zhou H , Thompson WW , Viboud CG , et al. Hospitalizations associated with influenza and respiratory syncytial virus in the United States, 1993–2008. Clin Infect Dis. 2012;54(10):1427‐1436. 22495079 10.1093/cid/cis211 PMC3334364

[4] Lina B , Georges A , Burtseva E , et al. Complicated hospitalization due to influenza: results from the Global Hospital Influenza Network for the 2017–2018 season. BMC Infect Dis. 2020;20(1):465. 32615985 10.1186/s12879-020-05167-4 PMC7330273

[5] Centres for Disease Control and Prevention . Estimates of deaths associated with seasonal influenza – United States, 1976–2007. MMWR Morb Mortal Wkly Rep. 2010;59(33):1057‐1062. 20798667

[6] Govaert TM , Dinant GJ , Aretz K , Masurel N , Sprenger MJ , Knottnerus JA . Adverse reactions to influenza vaccine in elderly people: randomised double blind placebo controlled trial. BMJ. 1993;307(6910):988‐990. 8241913 10.1136/bmj.307.6910.988 PMC1679213

[7] Vaccines against influenza WHO position paper – November 2012. Wkly Epidemiol Rec. 2012;87(47):461‐476. 23210147

[8] Osterholm MT , Kelley NS , Sommer A , Belongia EA . Efficacy and effectiveness of influenza vaccines: a systematic review and meta‐analysis. Lancet Infect Dis. 2012;12(1):36‐44. 22032844 10.1016/S1473-3099(11)70295-X

[9] Demicheli V , Jefferson T , Di Pietrantonj C , et al. Vaccines for preventing influenza in the elderly. Cochrane Database Syst Rev. 2018;2(2):Cd004876. 16856068 10.1002/14651858.CD004876.pub2

[10] Rondy M , El Omeiri N , Thompson MG , Levêque A , Moren A , Sullivan SG . Effectiveness of influenza vaccines in preventing severe influenza illness among adults: a systematic review and meta‐analysis of test‐negative design case‐control studies. J Infect. 2017;75(5):381‐394. 28935236 10.1016/j.jinf.2017.09.010 PMC5912669

[11] Haq K , McElhaney JE . Immunosenescence: influenza vaccination and the elderly. Curr Opin Immunol. 2014;29:38‐42. 24769424 10.1016/j.coi.2014.03.008

[12] Schaffner W , van Buynder P , McNeil S , Osterhaus A . Seasonal influenza immunisation: strategies for older adults. Int J Clin Pract. 2018;72(10):e13249. 30216647 10.1111/ijcp.13249

[13] Carrat F , Flahault A . Influenza vaccine: the challenge of antigenic drift. Vaccine. 2007;25(39–40):6852‐6862. 17719149 10.1016/j.vaccine.2007.07.027

[14] Calabro S , Tortoli M , Baudner BC , et al. Vaccine adjuvants alum and MF59 induce rapid recruitment of neutrophils and monocytes that participate in antigen transport to draining lymph nodes. Vaccine. 2011;29(9):1812‐1823. 21215831 10.1016/j.vaccine.2010.12.090

[15] Khurana S , Verma N , Yewdell JW , et al. MF59 adjuvant enhances diversity and affinity of antibody‐mediated immune response to pandemic influenza vaccines. Sci Transl Med. 2011;3(85):85ra48. 10.1126/scitranslmed.3002336 PMC3501657 21632986

[16] Frey SE , Reyes MR , Reynales H , et al. Comparison of the safety and immunogenicity of an MF59®‐adjuvanted with a non‐adjuvanted seasonal influenza vaccine in elderly subjects. Vaccine. 2014;32(39):5027‐5034. 25045825 10.1016/j.vaccine.2014.07.013

[17] Ansaldi F , Zancolli M , Durando P , et al. Antibody response against heterogeneous circulating influenza virus strains elicited by MF59‐ and non‐adjuvanted vaccines during seasons with good or partial matching between vaccine strain and clinical isolates. Vaccine. 2010;28(25):4123‐4129. 20433807 10.1016/j.vaccine.2010.04.030

[18] Domnich A , Arata L , Amicizia D , Puig‐Barberà J , Gasparini R , Panatto D . Effectiveness of MF59‐adjuvanted seasonal influenza vaccine in the elderly: a systematic review and meta‐analysis. Vaccine. 2017;35(4):513‐520. 28024956 10.1016/j.vaccine.2016.12.011

[19] Coleman BL , Sanderson R , Haag MD , McGovern I . Effectiveness of the MF59‐adjuvanted trivalent or quadrivalent seasonal influenza vaccine (FLUAD) among adults aged 65 or older, a systematic review and meta‐analysis. York, UK: PROSPERO; 2020. 10.1111/irv.12871 PMC8542957 34081398

[20] Sterne JA , Hernán MA , Reeves BC , et al. ROBINS‐I: a tool for assessing risk of bias in non‐randomised studies of interventions. BMJ. 2016;355:i4919. 27733354 10.1136/bmj.i4919 PMC5062054

[21] GRADE working group . GRADE handbook: handbook for grading the quality of evidence and the strength of recommendations using the GRADE approach. In: Schünemann H, Brożek J, Fuyatt G, Oxman A, editors.; 2013.

[22] Higgins JPT , Thomas J , Chandler J , et al. Cochrane Handbook for Systematic Reviews of Interventions (2nd edn.). Chinchester, UK: John Wiley & Sons; 2019.

[23] Dekkers OM , Vandenbroucke JP , Cevallos M , Renehan AG , Altman DG , Egger M . COSMOS‐E: guidance on conducting systematic reviews and meta‐analyses of observational studies of etiology. PLoS Med. 2019;16(2):e1002742‐e. 30789892 10.1371/journal.pmed.1002742 PMC6383865

[24] Lafond KE , Tam JS , Bresee JS , Widdowson M‐A . International meeting on influenza vaccine effectiveness, 3–4 December 2012, Geneva, Switzerland. Vaccine. 2014;32(49):6591‐6595. 25446822 10.1016/j.vaccine.2014.09.069 PMC5733129

[25] Bella A , Gesualdo F , Orsi A , et al. Effectiveness of the trivalent MF59 adjuvated influenza vaccine in preventing hospitalization due to influenza B and A(H1N1)pdm09 viruses in the elderly in Italy, 2017–2018 season. Expert Rev Vaccines. 2019;18(6):671‐679. 31159616 10.1080/14760584.2019.1627206

[26] Bellino S , Bella A , Puzelli S , et al. Moderate influenza vaccine effectiveness against A(H1N1)pdm09 virus, and low effectiveness against A(H3N2) subtype, 2018/19 season in Italy. Expert Rev Vaccines. 2019;18(11):1201‐1209. 31674847 10.1080/14760584.2019.1688151

[27] Pebody R , Whitaker H , Zhao H , et al. Protection provided by influenza vaccine against influenza‐related hospitalisation in >=65 year olds: early experience of introduction of a newly licensed adjuvanted vaccine in England in 2018/19. Vaccine. 2020a;38(2):173‐179. 31653528 10.1016/j.vaccine.2019.10.032

[28] Pebody RG , Whitaker H , Ellis J , et al. End of season influenza vaccine effectiveness in primary care in adults and children in the United Kingdom in 2018/19. Vaccine. 2020b;38(3):489‐497. 31685296 10.1016/j.vaccine.2019.10.071

[29] Van Buynder P , Konrad S , Van Buynder J , et al. The comparative effectiveness of adjuvanted and unadjuvanted trivalent inactivated influenza vaccine (TIV) in the elderly. Vaccine. 2013a;31(51):6122‐6128. 23933368 10.1016/j.vaccine.2013.07.059

[30] Van Buynder P , Konrad S , Van Buynder J , Ramler G . The comparative effectiveness of adjuvanted and unadjuvanted trivalent inactivated influenza vaccine in the elderly [poster]. OPTIONS VIII. Cape Town, SA: 2013b. 10.1016/j.vaccine.2013.07.059 23933368

[31] Public Health England . Surveillance of influenza and other respiratory viruses in the UK: winter 2019 to 2020. In: Section IS , ed. London, GB: Public Health England; 2020:69. https://assets.publishing.service.gov.uk

[32] Public Health England . Surveillance of influenza and other respiratory viruses in the UK: winter 2018 to 2019. In: Immunisation and Countermeasures Division NIS , Public Health England , ed. London, England: Public Health England; 2019:57.

[33] Puig‐Barbera J , Diez‐Domingo J , Perez Hoyos S , Belenguer Varea A , Gonzalez VD . Effectiveness of the MF59‐adjuvanted influenza vaccine in preventing emergency admissions for pneumonia in the elderly over 64 years of age. Vaccine. 2004;23(3):283‐289. 15530669 10.1016/j.vaccine.2004.07.017

[34] Puig‐Barberà J , Díez‐Domingo J , Varea ÁB , et al. Effectiveness of MF59‐adjuvanted subunit influenza vaccine in preventing hospitalisations for cardiovascular disease, cerebrovascular disease and pneumonia in the elderly. Vaccine. 2007;25(42):7313‐7321. 17889411 10.1016/j.vaccine.2007.08.039

[35] Gasparini R , Amicizia D , Lai PL , Rossi S , Panatto D . Effectiveness of adjuvanted seasonal influenza vaccines (Inflexal V and Fluad) in preventing hospitalization for influenza and pneumonia in the elderly: a matched case‐control study. Hum Vaccin Immunother. 2013;9(1):144‐152. 23143775 10.4161/hv.22231 PMC3667930

[36] Spadea A , Unim B , Colamesta V , et al. Is the adjuvanted influenza vaccine more effective than the trivalent inactivated vaccine in the elderly population? Results of a case‐control study. Vaccine. 2014;32(41):5290‐5294. 25087677 10.1016/j.vaccine.2014.07.077

[37] Lapi F , Marconi E , Simonetti M , et al. Adjuvanted versus nonadjuvanted influenza vaccines and risk of hospitalizations for pneumonia and cerebro/cardiovascular events in the elderly. Expert Rev Vaccines. 2019;18(6):663‐670. 31155968 10.1080/14760584.2019.1622418

[38] Fabiani M , Volpe E , Faraone M , Bella A , Pezzotti P , Chini F . Effectiveness of influenza vaccine in reducing influenza‐associated hospitalizations and deaths among the elderly population; Lazio region, Italy, season 2016–2017. Expert Rev Vaccines. 2020;19(5):479‐489. 32237925 10.1080/14760584.2020.1750380

[39] Izurieta HS , Chillarige Y , Kelman J , et al. Relative effectiveness of cell‐cultured and egg‐based influenza vaccines among elderly persons in the United States, 2017–2018. J Infect Dis. 2019;220(8):1255‐1264. 30561688 10.1093/infdis/jiy716

[40] Izurieta HS , Chillarige Y , Kelman J , et al. Relative effectiveness of influenza vaccines among the U.S. elderly, 2018‐19. J Infect Dis. 2020;222(2):278‐287. 32100009 10.1093/infdis/jiaa080

[41] Mannino S , Villa M , Apolone G , et al. Effectiveness of adjuvanted influenza vaccination in elderly subjects in northern Italy. Am J Epidemiol. 2012;176(6):527‐533. 22940713 10.1093/aje/kws313 PMC3447603

[42] Cocchio S , Gallo T , Del Zotto S , et al. Preventing the risk of hospitalization for respiratory complications of influenza among the elderly: is there a better influenza vaccination strategy? A retrospective population study. Vaccines. 2020;8(3):344. 10.3390/vaccines8030344 PMC7564213 32605238

[43] van Aalst R . Relative VE of HD vs adjuvanted influenza vaccine: a retrospective cohort study [abstract 10499]. OPTIONS X. Signapore; 2019.

[44] Pelton S , Divino V , Shah D , et al. Evaluating the relative vaccine effectiveness of adjuvanted trivalent influenza vaccine compared to high dose trivalent and other egg‐based influenza vaccines among older adults in the US during the 2017–2018 influenza season. Vaccines. 2020;8(3):2017‐2018. 10.3390/vaccines8030446 PMC7563546 32784684

[45] Divino V , Mould‐Quevedo J , DeKoven M , Jiang M , Krishnarajah G . Hospitalization encounters following vaccination with adjuvanted trivalent influenza vaccine compared to egg‐based trivalent high‐dose, egg‐based quadrivalent and trivalent vaccines among the US elderly using claims data [poster]. OPTIONS X. Singapore. 2019a.

[46] Divino V , Mould‐Quevedo J , Jiang M , DeKoven M , Krishnarajah G . Real‐world outcomes of adjuvanted trivalent influenza vaccine compared to egg‐based trivalent high‐dose, egg‐based quadrivalent and trivalent vaccines among the US elderly during 2016–2018 flu seasons using claims data [poster]. OPTIONS X; Singapore. 2019b.

[47] Boikos C , Fischer L , O'Brien D , Vasey J , Sylvester G , Mansi J . Relative effectiveness of aTIV versus TIVe, QIVe and HD‐TIV in preventing influenza‐related medical encounters during the 2017–2018 and 2018–2019 influenza seasons in the US [poster]. National Foundation for Infectious Diseases. Virtual: 2020.

[48] McConeghy KW , Davidson HE , Han L , et al. Can adjuvanted influenza vaccine given as standard of care reduce the risk for influenza outbreaks in nursing homes: evidence from a cluster‐randomized trial of 823 nursing homes [abstract]. European Congress of Clinical Microbiology and Infectious Diseases. Virtual. 2020.

[49] Gravenstein S , Davidson HE , McConeghy KW , et al. Effectiveness of adjuvanted vs. non‐adjuvanted influenza vaccine in US nursing homes [poster]. Baltimore, MD; National Foundation for Infectious Diseases: 2019.

[50] Gravenstein S , Davidson HE , McConeghy KW , et al. A cluster‐randomized trial of adjuvanted trivalent influenza vaccine vs. standard dose in US nursing homes [abstract]. IDWeek. San Diego, USA; Open Forum Infectious Diseases: 2018:S296.

[51] van Aalst R , Gravenstein S , Mor V , et al. Comparative effectiveness of high dose versus adjuvanted influenza vaccine: a retrospective cohort study. Vaccine. 2020;38(2):372‐379. 31606249 10.1016/j.vaccine.2019.09.105

[52] Boikos C . Poster clarification [email]. In: Coleman BL, ed. 2020.

[53] De Smedt T , Merrall E , Macina D , Perez‐Vilar S , Andrews N , Bollaerts K . Bias due to differential and non‐differential disease‐ and exposure misclassification in studies of vaccine effectiveness. PLoS ONE. 2018;13(6):e0199180. 29906276 10.1371/journal.pone.0199180 PMC6003693

