# Supplementary material for: Effectiveness of the MF59‐adjuvanted trivalent or quadrivalent seasonal influenza vaccine among adults 65 years of age or older, a systematic review and meta‐analysis
Source: Influenza Other Respir Viruses. 2021 Jun 3;15(6):813–23. doi: 10.1111/irv.12871 (PMC8542957; doi:10.1111/irv.12871)
Supplement: Supplementary file 1 — Table S1‐S3 [file IRV-15-813-s001.docx]

**Supplementary Tables**

**Effectiveness of the MF59-adjuvanted trivalent or quadrivalent seasonal influenza vaccine among adults 65 years of age or older, a systematic review and meta-analysis**

Coleman BL, Sanderson R, Haag MDM, McGovern I

**Table S1:**

##### Search Terms: FLUAD terms (i.e., fluad or MF59 or MF?59 or aTIV or aQIV or chiromas or gripguard or Influpozzi Adiuvato or allV3 or allV4), influenza virus terms (i.e., influenza vaccines, Influenza, Human, quadrivalent, influenza a virus/ or influenza a virus, h1n1 subtype/ or influenza a virus, h3n2 subtype, influenzavirus b/ influenza b virus, (influenza* or flu).ti, ab, kf) vaccine terms (i.e., vaccin* or immuni* or innoculat*.ti,ab,kf) and types of vaccines (i.e., adjuvant* or squalene* or emulsion*.mp.).

##### Limits: Limited to humans and excluding animal studies (e.g., rats, etc.). Limited to “1997-Current”. Limited to English, French, Italian or Spanish.

##### Note that we used the Boolean NOT operator to exclude animal studies and publication types (e.g., phase 1 clinical trials, placebos, case reports, letters or historical articles). We refined and expanded search terms as required to ensure the retrieval of sentinel references (e.g., specifying rat or rats as excluded animals instead of rat*.

Table: Search Terms

| **DATABASE and search terms** | | **Type/ Comment** |
| --- | --- | --- |
| **MEDLINE** | | |
| 1 | (fluad* or MF59* or MF?59* or aTIV or aQIV or chiromas or gripguard or Influpozzi Adiuvato or aIIV3* or aIIV4*).mp. | FLUAD terms |
| 2 | influenza vaccines/ |  |
| 3 | Influenza, Human/ |  |
| 4 | influenza a virus/ or influenza a virus, h1n1 subtype/ or influenza a virus, h3n2 subtype/ |  |
| 5 | influenzavirus b/ or influenza b virus/ |  |
| 6 | (influenza* or flu).ti,ab,kf. |  |
| 7 | or/3-6 | Influenza virus terms |
| 8 | exp vaccines/ |  |
| 9 | (vaccin* or immuni* or innoculat*).ti,ab,kf. |  |
| 10 | 8 or 9 | Vaccine terms |
| 11 | 2 or (7 and 10) | Influenza vaccine terms |
| 12 | (adjuvant* or squalene* or emulsion*).mp. |  |
| 13 | 11 and 12 | Type of vaccines |
| 14 | 1 or 13 | Base clinical set |
| 15 | exp Animals/ |  |
| 16 | exp Humans/ |  |
| 17 | 15 not (15 and 16) | Animal not human results |
| 18 | (mouse or mice or rat or rats or bird or avian or poultry or rabbit or rabbits or cat or cats or dogs or ferret or ferrets or pig or pigs or hamster or hamsters or monkey or monkeys or chimp or chimps or equine or canine or canines or primate or primates).mp. |  |
| 19 | 14 not (17 or 18) | Human results |
| 20 | pandemics/ or pandemic*.ti,ab,kf. |  |
| 21 | season*.ti,ab,kf. |  |
| 22 | 20 not (20 and 21) [*** non-seasonal flu results****] |  |
| 23 | 19 not 22 | Non-seasonal flu results |
| 24 | (clinical trial, phase i or clinical trial, phase ii).pt. |  |
| 25 | Placebos/ |  |
| 26 | placebo*.ti,ab,kf. |  |
| 27 | case reports.pt. |  |
| 28 | (historical article or letter).pt. |  |
| 29 | or/24-28 | Publication type terms |
| 30 | 23 not 29 | Publication types removed |
| 31 | limit 30 to yr="1997 -Current" | Publication year limit |
| 32 | limit 31 to (english or french or italian or spanish) | Language limit – Final results |
| **Open Grey** | | |
| (fluad* or "MF59*" or "MF?59*" or aTIV or aQIV or chiromas or gripguard or "Influpozzi Adiuvato" or aIIV3* or aIIV4*) = 0 refs  influenza vaccin* AND (adjuvant* OR squalene* OR emuls*) = 8 refs – only 7 human studies | | |

Table S2: Study characteristics and results for adjusted absolute and relative vaccine effectiveness of adjuvanted trivalent inactivated vaccine

| **Study** | **Season** | **Country**  **(Setting)** | **Design**  **(EM)** | **Outcome** | **Compar-ator of interest** | **VE**  **(aTIV)** | **VE**  **(comparator of interest)** | **rVE** | **Cases** | **Controls** |
| --- | --- | --- | --- | --- | --- | --- | --- | --- | --- | --- |
| Van  Buynder(a)^30^ | 2011/12 | Canada  (GP visit) | TND  (OR) | Lab confirmed  -All types | No vaccine  TIV | 58.1 (4.9, 81.5) | -1.7 (-139, 56.8) | 63 (4, 86) | 61 | 159 |
| Van Buynder(b)^31^ | 2012/13 | Canada  (GP visit)  (Hospital) | TND (OR) | Lab confirmed  -All types | No vaccine | 39.3 (13.8, 57.3)  75.6 (55.3, 86.7) |  |  | 385  283 | 528  630 |
| Bella^26^ | 2017/18 | Italy  (Hospital) | TND  (OR) | Lab confirmed  -All types  -H1N1  -B (any) | No vaccine | 48.3 (18.7, 67.2)  67.5 (8.9, 88.4)  44.5 (8.5, 66.3) |  |  | 118  23  91 | 384 |
| Pebody (a)^29^ | 2018/19 | UK  (Hospital) | TND  (OR) | Lab confirmed  -All types  -H1N1  -H3N2 | No vaccine  TIV/QIV | 53.8 (39.8, 64.5)  65.9 (50.6, 76.4)  39.5 (4.8, 61.5) | 34.4 (-72.2, 75)  64.9 (‑65.9, 92.6)  ‑5.2 (-336, 74.7) | 29.6 (-83.4, 73.0)  2.8 (-358, 79.4)  42.5 (-134, 85.8) | 377  160  108 | 880 |
| Peabody (b)^28,33^ | 2018/19 | UK  (GP visit) | TND  (OR) | Lab confirmed  -All types  -A (any) | No vaccine | 62.0 (3.4, 85.0)  62.0 (3.4, 85.0) |  |  | 57  56 | 237 |
| Bellino^27^ | 2018/19 | Italy  (GP visit) | TND  (OR) | Lab confirmed  -A (any) | No vaccine  QIV | -1.1 (-122, 57.8) | 3.1 (-1.6, 54.3) |  | 47 | 72 |
| Public Health England^32^ | 2019/20 | UK  (GP visit) | TND  (not stated) | Lab-confirmed  -All types  -A(H3N2) | No vaccine | 16.2 (-58.7, 55.7)  8.6 (-81.9, 54.1) |  |  | Not stated | Not stated |
| Spadea^38^ | 2011/12 | Italy | CC  (OR) | Hospitalized  ‑influenza/  pneumonia | No vaccine | 49 (30, 60) |  |  | 365 | 1227 |
| Puig-Barberà (a)^35^ | 2002/03 | Spain | CC  (OR) | Hospitalized  ‑influenza/  pneumonia | No vaccine | 48 (20, 66) |  |  | 290 | 525 |
| Puig-Barberà (b)^34^ | 2004/05 | Spain | CC  (OR) | Hospitalized  ‑influenza/  pneumonia | No vaccine | 69 (29, 86) |  |  | 198 | 321 |
| Gasparini^37^ | 2010/11 | Italy | CC  (OR) | Hospitalized  ‑influenza/  pneumonia | No vaccine | 87.8 (0, 98.9) | 95.2 (62.8, 99.4) |  | 187 | 187 |
| Lapi^36^ | 2002/03 to  2016/17 | Italy | CC | Hospitalized  ‑pneumonia/  cerebrovascular/  cardiovascular | TIV |  |  | 39 (4, 61) | 103 | 748 |
| Mannino^42^ | 2006/07 to 2008/09 | Italy | Cohort  (RR) | Hospitalization  (peak weeks)  ‑influenza/  pneumonia | TIV |  |  | 25 (2, 43) | 225 | 164254 |
| Cocchio^43^ | 2011/12 to  2016/17 | Italy | Cohort  (OR) | Hospitalization  ‑influenza/  pneumonia | TIV  (possibly some QIV) |  |  | 33 (25, 41) | 3176 | 479397 |
| Fabiani^39^ | 2016/17 | Italy | Cohort  (HR) | Death or hospitalization  ‑influenza/  pneumonia/ respiratory condition/ cardiovascular | TIV  QIV | 16 (12, 19) | 13 (9, 16)  16 (12, 20) |  | 305319  135502 | 609994 |
| Van Aalst^52^ | 2016/17 | USA | Cohort  (RR) | Hospitalization  ‑respiratory  -cardio-respiratory | HD TIV |  |  | -14.9 (-47.1, 5.9)  -14.9 (-29.9, -2.4) | 513  1584 | 15169  45738 |
| Van Aalst^52^ | 2017/18 | USA | Cohort  (RR) | Hospitalization  ‑respiratory  -cardio-respiratory | HD TIV |  |  | -13.6 (-26.6, -2.1)  -6.4 (-12.4, -0.6) | 3293  9918 | 21260  62269 |
| Izurieta^40^ | 2017/18 | USA | Cohort  (RR) | 1) Hospitalization or ED consult  2) Office visit  -influenza | TIV  QIV  HD TIV  TIV  QIV  HD TIV |  |  | 3.6 (0.7, 6.4)  3.9 (1.4, 6.3)  -5.6 (-7.9, -3.4)  -11.9 (-15.9, -8.1)  -6.6 (-9.7, -3.5)  -7.3 (-9.8, -4.8) | 93182  71917 | 13504092  13504092 |
| Pelton^45,47^ | 2017/18 | USA | Cohort  (RR) | 1) Hospitalization or ED or GP consult  2) Office visit  -influenza | TIV  QIV  HD TIV  TIV  QIV  HD TIV |  |  | 11.2 (2.3, 19.4)  8.6 (1.2, 15.6)  3.2 (-3, 9)^ŧ^  25.0 (17.0, 32.2)  36.3 (31.0, 41.2)  16.6 (10.8, 22.0) |  | 1822946  1822946 |
| Boikos^48^ | 2017/18 | USA | Cohort  (OR) | Hospitalization or ED or GP consult  -influenza | TIV  QIV  HD TIV |  |  | 7.5 (4.2, 10.6)  18.2 (15.8, 20.5)  7.7 (2.3, 12.8) |  | 5201573 |
| Izurieta^41^ | 2018/19 | USA | Cohort  (RR) | Hospitalization or ED consult  -influenza | QIV  HD TIV |  |  | 6.5 (1.5, 11.3)  2.9 (0, 5.7) | 34274 | 12277214 |
| Boikos^48^ | 2018/19 | USA | Cohort  (OR) | Hospitalization or ED or GP consult  -influenza | TIV  QIV  HD TIV |  |  | 25.6 (18.2, 32.2)  27.8 (25.7, 29.9)  6.9 (3.1, 10.6) |  | 5981125 |
| Gravenstein^50,51^ | 2016/17 | USA | C-RCT (HR) | Hospitalization  ‑influenza/  pneumonia | TIV |  |  | 21 (4, 35) | 1730 | 48282 |
| McConeghy^49^ | 2016/17 | USA | C-RCT  (RR) | Outbreak  -influenza-like illness | TIV |  |  | 22 (1, 37) | 777 | 46 |

*Other rVE estimated include: asthma/chronic obstructive pulmonary disease/bronchial, coronary artery, myocardial infarction, congestive heart failure, cerebrovascular, stroke, and other respiratory hospitalizations/ED visits

ŧ confidence interval estimated from figures

aTIV: adjuvanted trivalent inactivated vaccine; CC: case-control; C-RCT: cluster randomized control trial; ED: emergency department; EM: effect measure; GP: general practitioner/outpatient; HD TIV: high dose TIV; HR: hazard ratio; ID TIV: intradermal TIV; OR: odds ratio; QIV: quadrivalent inactivated vaccine; RR: relative risk/rate ratio; rVE: relative vaccine effectiveness; TIV: trivalent inactivated vaccine; VE: vaccine effectiveness;

NOTES:
Iob et al.^55^ conducted a non-randomized cluster observational study during the 1998/99 season in long-term care facilities in Italy. The facilities vaccinated their residents with either aTIV or TIV and followed them for episodes of ILI. This study is not being included in a meta-analyses due to the inclusion of residents as young as 23 years of age (3.6% were younger than 65 years of age). The unadjusted (crude) relative VE was 36% (18, 46) in preventing ILI in these residents.

Mira-Iglesias et al.^56^ conducted a test-negative study in Spain in the 2017/18 season in hospitalized patients who were 60 years of age or older (18.2% of cases and 20.4% of controls were 60-69 years old), which made this study ineligible for the review. The adjusted VE estimates for aTIV were 9.97% (-24.43, 34.86) against any/all types of influenza, 34.38% (-34.58, 68.00) against influenza A(H1N1pdm) and -23.93% (-87.94, 18.28) against influenza A(H3N2). No estimate was given for influenza B.

Table S3: Assessment of risk of bias using ROBINS-I tool

| **Study** | **Design** | **Risk of confounding** | **Risk of**  **selection bias** | **Risk of**  **Misclassify-cation of exposure** | **Risk of deviation from intended vaccine** | **Risk of missing data** | **Risk of misclass-ification of outcomes** | **Risk of reporting bias** | **Overall risk of bias** |
| --- | --- | --- | --- | --- | --- | --- | --- | --- | --- |
| Bella^26^ | Test negative | Low | Moderate | Moderate | Low | Moderate | Low | Low | **Moderate** |
| Pebody(a)^29^ | Test negative | Low | Moderate | Moderate | Low | Moderate | Low | Low | **Moderate** |
| Pebody(b)^28,33^ | Test negative | Low | Moderate | Moderate | Low | Moderate | Low | Low | **Moderate** |
| Van Buynder(a)^30^ | Test negative | Low | Moderate | Moderate | Low | Moderate | Low | Low | **Moderate** |
| Van Buynder(b)^31^ | Test negative | Low | Moderate | Moderate | Low | Moderate | Low | Low | **Moderate** |
| Bellino^27^ | Test negative | Low | Moderate | Moderate | Low | Moderate | Low | Low | **Moderate** |
| PHE^32^ | Test negative | No info | Moderate | Moderate | Low | Moderate | Low | Low | **Moderate** |
| Gasparini^37^ | Case-control | Moderate | Serious | Moderate | Low | Moderate | Moderate | Low | **Serious** |
| Spadea^38^ | Case-control | Low | Moderate | Moderate | Low | Moderate | Moderate | Low | **Moderate** |
| Puig-Barberà^35^ | Case-control | Low | Moderate | Moderate | Low | Moderate | Moderate | Low | **Moderate** |
| Puig-Barberà^34^ | Case-control | Low | Moderate | Moderate | Low | Moderate | Moderate | Low | **Moderate** |
| Lapi^36^ | Case-control | Low | Moderate | Moderate | Low | Moderate | Moderate | Moderate | **Moderate** |
| Fabiani^39^ | Cohort | Moderate | Low | Low | Low | Low | Moderate | Low | **Moderate** |
| Izurieta^40^ | Cohort | Low | Low | Low | Low | Low | Moderate | Moderate | **Moderate** |
| Izurieta^41^ | Cohort | Low | Low | Low | Low | Low | Moderate | Moderate | **Moderate** |
| Mannino^42^ | Cohort | Moderate | Moderate | Low | Low | Low | Moderate | Moderate | **Moderate** |
| Cocchio^43^ | Cohort | Moderate | Low | Low | Low | Moderate | Moderate | Moderate | **Moderate** |
| Van Aalst^52^ | Cohort | Low | Low | Low | Low | Moderate | Moderate | Moderate | **Moderate** |
| Pelton^45,46^ | Cohort | Low | Low | Low | Low | Low | Moderate | Moderate | **Moderate** |
| Pelton^45,47^ | Cohort | Low | Low | Low | Low | Low | Moderate | Moderate | **Moderate** |
| Boikos^48^ | Cohort | Low | Low | Low | Low | Low | Moderate | Moderate | **Moderate** |
| Gravenstein^50,51^ | Cluster-RCT | Low | Low | Low | Low | Moderate | Moderate | Moderate | **Moderate** |
| McConeghy^49^ | Cluster-RCT | Low | Low | Low | Low | Moderate | Moderate | Moderate | **Moderate** |

Info: information; RCT: randomized control trial
